# Bridging Gaps: Provider Perspectives on Integrating Systems for Health Equity

**DOI:** 10.3390/ijerph22040550

**Published:** 2025-04-02

**Authors:** Brittany R. Schuler, Stacey L. Shipe, Astrid Uhl, Samantha Smith, LaShanta Majeed, Nicole O’Reilly, Cheri Carter, Bradley N. Collins

**Affiliations:** 1 School of Social Work, College of Public Health, Temple University, 1301 Cecil B. Moore Ave., Ritter Annex 549, Philadelphia, PA 19122, USA; astrid.uhl@temple.edu (A.U.); cheri.carter@temple.edu (C.C.); 2 Social Work, Binghamton University, State University of New York, University Downtown Center, 67 Washington St., Binghamton, NY 13902, USA; sshipe@binghamton.edu; 3 Bethesda Project, KIPP Dubois Collegiate Academy High School, Philadelphia, PA 19131, USA; ssamantha26@hotmail.com; 4 Turning Points for Children, 415 S 15th St., Philadelphia, PA 19146, USA; lasmajeed@gmail.com; 5 Social Work, McDaniel College, Westminster, MD 21157, USA; nicole.oreilly@mcdaniel.edu; 6 Department of Social and Behavioral Sciences, College of Public Health, Temple University, 1301 Cecil B. Moore Ave., Philadelphia, PA 19122, USA; bradley.collins@temple.edu

**Keywords:** social work, health and mental health professionals, community organizations, low socioeconomic status, qualitative, children and families, qualitative

## Abstract

Health equity is shaped by multiple factors intersecting with service delivery in community-based organizations (CBOs). Providers in under-resourced areas are often the first point of contact for families seeking child development, mental health, and behavioral support. However, system-level barriers hinder service delivery and access. This study explores provider perspectives to identify barriers and inform system-level changes that promote equity in child and family health. Using a narrative qualitative design, in-depth interviews were conducted with 21 health and mental health professionals from child- and family-serving CBOs. Guided by ecological and strengths-based frameworks, interviews examined provider insights on challenges, strengths, and supports affecting service delivery. Key themes emerged across macro (rights-based policies, racism/oppression), community (environmental impacts, social cohesion), organizational (secondary stress, system fragmentation, provider supports), and family levels (basic needs, parenting support, service access). Findings highlight the need for a multilevel approach that prioritizes rights-based policies, strengthens community cohesion, and improves system integration. Enhancing CBO capacity to address these determinants could advance equity-oriented service delivery and mitigate structural barriers that perpetuate health disparities.

## 1. Introduction

Unaddressed social needs, such as food and housing insecurity, combined with chronic forms of economic hardship can negatively impact child physical and mental health [1,2] and parent mental health. These same social needs can influence parenting factors that undermine developmental health and health behaviors [1,3] as well as family functioning [4]. Without more effective, coordinated strategies to address these social needs, pervasive health inequities in underserved communities will persist. A more expanded understanding of the multiple determinants linked to the mitigation of unmet social needs and its consequences on health is critical to informing health and social care providers and system-level efforts to promote health equity. For children in economically underserved communities, health and mental health professionals in community-based organizations (CBOs) are often the first and only point of contact for many families seeking or receiving services to support basic social needs (e.g., housing, civil rights), child development, caregiver/child mental and behavioral health, or physical health (e.g., food and nutrition security). Health and mental health professionals delivering such services have invaluable perspectives on child and family health and wellbeing. Evidence suggests systems coordination is important [5,6]. Efforts to improve systems coordination as well as program uptake, engagement, and effectiveness could be advanced with a more in-depth understanding as to how providers could facilitate coordinated operations and implement strategies to address multilevel barriers to promoting health equity [5,6]. Multiple levels of barriers exist across the ecology within which social service agencies operate in under-resourced communities that affect their programs’ impact and the degree to which families seeking service access and remain engaged in those programs [7,8]. However, research on health and mental health professionals working within community networks is highly limited.

Ecological systems theory aims to understand the interacting relationships between individuals and the broader interacting community, social, economic, and political forces that influence overall health and wellbeing [9,10]. For the purposes of this study, ecological levels of determinants were defined as (1) macro–broader social, cultural, and political factors that interact with other levels to affect child and family health and wellbeing; (2) community–formal and informal social structures that directly impact child/family-serving organizations and that indirectly operate to impact families; (3) organizational–the system of interlinked organizations, community groups, and institutions that interact with macro, community, and family levels to impact child or family health and wellbeing; and (4) family–factors that have direct impacts on or contact with the child/family or their immediate, proximal environments. In the context of organizational systems situated within urban communities that impact family systems, ecological systems theory incorporates the environmental level of influence (e.g., health systems, community factors), including multilevel sources of strength and support in addition to risk factors [11]. Within this ecology exists multiple levels of determinants that interact with one another in undermining social service program access, engagement, and effectiveness. The interaction between system-level barriers and personal-level barriers creates a set of circumstances that inhibits service delivery and service access [12]. However, individual CBOs or sets of organizations are not typically set up to address the results of this interaction [6]. Moreover, most families in under-resourced communities require multiple services that no single system or agency provides. Most existing approaches are fragmented and geared towards changing individuals’ behaviors, placing responsibility on the parent or child to follow up with referrals and use isolated downstream approaches (i.e., focus of responsibility is on micro individual change) rather than midstream (i.e., change in a community or group) and upstream, (i.e., macro or system level) approaches that address underlying drivers of health disparities [13].

Improving linkages across separate systems of care could overcome the challenges of multiple service needs and barriers to program access, engagement, and effectiveness if not better address interacting multilevel barriers that undermine the impact of social services in underserved communities. Existing research in healthcare settings found that creating coordinated systems of care to connect patients across community-based and social service assistance programs can help to increase service access and improve health and mental health outcomes [14,15,16], demonstrating how attending to fundamental social needs could help to moderate socioeconomic-driven disparities [17]. Understanding the perspectives of health and mental health professionals working within one system network of community agencies serving low-income children and families can provide important information on the common underlying socioeconomic drivers of disparity or equity in child physical and mental health development, information that is not currently well documented.

Prior research highlights the significant impact of unaddressed social needs—such as food and housing insecurity—on child and parent health, family functioning, and broader health inequities. While systems coordination has been identified as a critical factor in mitigating these challenges, limited research exists on the perspectives of health and mental health professionals working within community networks, particularly in how they navigate multilevel barriers to service access, engagement, and effectiveness. Our study directly addresses this gap by focusing on the experiences of professionals working within a coordinated system of community-based organizations (CBOs). Using ecological systems theory as a guiding framework, our research questions explore systemic, community, and organizational barriers and strengths, as well as facilitators that influence service delivery and engagement. Research questions are designed to provide a more in-depth understanding of how providers within a structured network perceive and address complex, multilevel determinants of health equity. By examining these perspectives, our study contributes to the existing literature by identifying actionable insights that could inform efforts to improve systems coordination and enhance CBOs’ capacity to address social determinants of health. The specific research questions were:

1. What are the major systemic, community, and organizational barriers to promoting better health/mental health for children and families?
2. What are the major systemic, community, and organizational strengths that help promote family health/mental health?
3. What are the major systemic, community, and organizational facilitators to health/mental health utilized by families?

## 2. Materials and Methods

### 2.1. Approach

A cross-sectional, narrative research approach was used to collect and analyze accounts of experiences as described by health and mental health professionals based on their lived and told stories [18,19]. Narrative research is an effective qualitative approach that can lead to enhanced delivery of care [18]. Through interviews, the narrative approach allows for in-depth exploration of personal experiences, meaning people assign to their perceptions or experiences and can provide insight into decisions involving treatment, screening, or various health practices, which can help guide how social care services are developed and provided [18,20].

### 2.2. Recruitment

Recruitment followed a purposive sampling approach [21]. To identify potential participants, the research team recruited from a local coalition comprising child and family serving organizations in addition to a research team compiled list of other related local CBOs in Philadelphia, Pennsylvania, a major city in the northeastern United States that is classified as a persistently impoverished area [22]. We intentionally identified health and mental health professionals with expertise in mental and behavioral health, food assistance, maternal and child health, and child welfare. After identifying sites for recruitment, the research team reached out to site directors to discuss the project and gain permission to recruit health and mental health professionals. Participants were eligible if they were health or mental health professionals with expertise in mental and behavioral health, food assistance, maternal and child health, or child welfare. Individuals who did not meet these professional criteria were excluded. No participants who met the inclusion criteria were excluded from the study. Following permission, information about the study was distributed through work-based electronic list-serves and flyers distributed by supervisors through list-serves. We asked participants to refer other health and mental health professionals in their social and professional networks using a secondary snowball sampling strategy [23]. Sample size was based on thematic saturation. Data collection continued until no new themes emerged from participant responses. Thematic saturation ensured that our sample size was sufficient to capture a comprehensive range of perspectives while maintaining methodological rigor. Institutional review board (IRB) approval was granted by the university’s review board.

### 2.3. Interview Measure

The Ecocultural Family Interview (EI) is a semi-structured interview guide that uses a mix of conversation and probing questions by trained interviewers [24]. The EI topics included four primary areas: (1) home and environment, (2) family health, (3) organizational-level factors, (4) and community supports and services. Content from these topic areas were then adapted to the circumstances of administrators and direct line health and mental health professionals working in child and family-serving CBOs. Specifically, adaptations were guided by the existing literature on the social determinants of health, emphasizing the impact of unaddressed social needs on child and family wellbeing. Additionally, input from key stakeholders, including health and mental health professionals working in community-based organizations, helped identify relevant topics such as health behaviors, health routine goals, child development, and available supports and services. The protocol was refined iteratively to ensure alignment with the ecological systems framework and to capture emerging themes related to service access, engagement, and coordination. Adaptations were made by the research team (principal investigator and MSW-level research assistant) and reviewed by a team of community stakeholders (5 MSW-level practitioners/administrators) to integrate current relevant topics regarding health behaviors and health routine goals, child development, and supports and services. The measure was piloted among a sample of health and mental health professionals and revised to reflect community-specific priorities for healthy child development and family functioning across four levels of the child’s ecology (see Table 1 for global questions and sample probing items). Interviewers provided participants with a brief and simple introduction of the primary EI topics, and health and mental health professionals were explicitly asked to consider how each topic played a role in service delivery and in the health and development of children and families they serve.

### 2.4. Data Analysis

All interviews were audio-recorded and transcribed verbatim. Analysis was conducted using qualitative analytic software (MaxQDA v.20) using an ongoing, iterative process following a systematic approach recommended for health research [25] and narrative research [26]. This approach included thematic analysis of the text in 3 steps: (1) reading for an overall understanding to identify overarching emergent themes within each primary topic, (2) applying a formal system of coding to a subset of transcripts to organize data and understand thematic linkages, followed by coding according to overall themes developed in step 1 until thematic saturation is reached, and (3) finalizing and applying the coding structure to the full set of transcripts [25]. Structural codes were developed for macro, community, organization, and family levels by staying very close to the text and describing its visible and apparent content [27]. This resulted in the development of sub-categories under the structural parent codes. These sub-categories were later organized in both concept-driven and data-driven categories, in which we tried to find the underlying meaning of the discussion [27]. Categories and sub-categories were redefined using an iterative process, creating new categories as appropriate [28]. Coding was conducted independently by the PI and a trained research assistant, with a third reviewer assisting with triangulation and final decision making. The steps for coding included reading each interview independent of coding. Once all interviews were read, the PI and research assistant coded the first three interviews to determine coder consistencies and inconsistencies. Based on these initial codes, the similarities and differences were discussed. This resulted in identifying a coding structure for the remaining interviews. Once all interviews were coded, final decisions were reviewed with an external team member with a PhD in Social Work. All three members of the team met in regular intervals to discuss coding decisions throughout data analysis.

### 2.5. Rigor and Vigor

Recommended strategies to enhance rigor and vigor of qualitative research are to promote credibility (i.e., internal validity) by assessing accuracy of the analysis by member checking. The process of sharing and discussing the analytic results as they unfold with participants who completed interviews helped to ensure the themes and discussion reflect what was intended [29]. Second, an expert consultant assisted with the data analysis and assessment of the quality of data analysis. Third, researchers took field notes and debriefed after interviews to discuss potential biases that the researcher brought to the study.

### 2.6. Reflexive Accounting

Reflexivity, or a person’s self-examination of personal beliefs and biases in the context of data collection and analysis, can strengthen the rigor of a study. Awareness of any influence that personal experiences may have on the research process, including race, gender, and social class, can allow for self-correction and elevate the critical thinking involved in decision making and post-study discussion and conclusions [30]. Interviewers completed field notes after each interview to document the context and setting of the interview, any difficulties or interruptions to the interview, and reflexive accounting of any personal biases or assumptions that may have affected the way they conducted the interview.

## 3. Results

This section may be divided by subheadings. It should provide a concise and precise description of the experimental results, their interpretation, as well as the experimental conclusions that can be drawn.

### 3.1. Sample Characteristics

A total of *n* = 21 interviews were conducted, lasting approximately 45 min on average (range: 30 to 60 min). Participants were 81% (*n* = 17) female and represented diverse racial and age groups (see Table 2). Participants held positions in various social service programs, including food assistance (*n* = 5), mental/behavioral health (*n* = 2), youth development (*n* = 1), child welfare (*n* = 10), advocacy and legal aid (*n* = 1), or other community programs for children and families (*n* = 2). Participants had diverse educational backgrounds, including social work (*n* = 4), education (*n* = 4), other human service or social science (*n* = 7; e.g., bachelor or master of science, master of health science, psychology), public health (*n* = 1), or another advanced degree (*n* = 5; e.g., law, political science). Participants also had varying levels of experience in the field, ranging from 1 to 44 years (*M* = 13.47, *SD* = 12.33). The experience of participants in their current role ranged from 1 to 29 years (*M* = 6.4, *SD* = 7.07).

### 3.2. Main Findings

As shown in Table 3, major themes that emerged from the analysis were organized according to the ecological systems framework reflecting multiple levels of influence and the four primary levels of the child’s social ecology: (1) macro level, (2) community level, (3) organization level, and (4) family level.

#### 3.2.1. Macro-Level Themes

The broader social, cultural, and political macro factors affecting child and family wellbeing included two subthemes; (a) rights-based policies and programming, primarily related to policies that support economic stability and affordable housing, and (b) macro-level policies or programs that have disproportionate effects on racial and ethnic minority populations.

Rights-based Policy and Programming. Providers thought working on and investing in anti-poverty policies, like employment/wage stability and housing stability were essential first steps in addressing disparities. Health and mental health professionals discussed the need for access to free quality healthcare, adequate and affordable food, good jobs, and adequate housing. One service provider described access to basic needs as “a miracle pill” for child and family wellbeing and prevention of child maltreatment. System-level interventions to promote economic security such as food and housing programs that “seriously address poverty” were referenced as a critical factor for preventing justice system involvement, child welfare system involvement, and family separations.

Racism and Oppression. This theme represents policies, programs, or practices described as alienating, stigmatizing, or oppressing racial and ethnic minority populations. Like many other major urban areas in the US, Philadelphia experiences a lot of challenges with racial disparities, and this intersects with poverty to make things more complicated for families. Racism and oppressive practices were referenced on multiple occasions by several participants. Examples include references to the over-reporting of Black and Hispanic families to the child welfare system, inappropriately using “drug tests as a proxy for parenting tests”, and fears parents have engaging in basic healthcare systems. This also included discussion of how health and mental health professionals manage bias when it shows up at the organizational level: “… and when you get called on it, you’re like ‘I ain’t biased, I ain’t biased.’ Yeah, but you are. Let’s talk about it. Let’s work through it because the families are suffering”. In addition, others described the structure of social services as not only inadequate but potentially harmful to families and children. The structure of existing services incites fears of being reported to child welfare or legal systems for investigation rather than hopes for supportive resources:

*The structural conditions that we’ve created and like that’s the design of the child welfare system we’re going to isolate individual families for like surveillance control and punishment and ignore that like we kind of created this system where they can’t find basic supports for the things that they need to just parent their children adequately.*

#### 3.2.2. Community-Level Themes

Community-level themes included aspects of the physical community environment and factors related to building opportunities for positive social interactions in the community that promote or detract from family wellbeing. The physical environment includes the physical structures, features, and facilities that make up the environments in which people live, work, or interact and the ways in which communal spaces are structured and used. Lack of proper infrastructure was referenced as being a major impediment to service access due to impacts on feelings of individual self-worth and safety. Health and mental health professionals referenced ways in which the built or physical environment surrounding families would benefit from infrastructures designed to promote health, such as safe, sanitary, accessible places for community engagement and interaction. One provider highlighted the effect the physical environment can have on family wellbeing, saying “you know if you live on a street where there’s constantly trash everywhere you feel like disrespected you feel like not worthy…”. Safety concerns were a barrier for families and health and mental health professionals alike, with one participant saying:

*‘I can’t go that REC Center. Because when I go over there, you know, I’ll get jumped, I’ll get shot.’ So I have parents who, even if they have the resources now, the park just got rebuilt, ‘I’m too afraid to take my kid outside.’*

Building Community. Building community was a subtheme of the community level that refers to social factors that support health or facilitate access to health-promoting resources related to community engagement practices, partnerships, or wraparound supports. Listening to the community, respecting community members as the experts, and creating safe spaces where people feel welcome to receive help from peers in non-judgmental ways were suggestions to improve community engagement. Health and mental health professionals described needing to “listen to everything…work side by side with people and listen to what they need and what they want” and seeing themselves as “just the facilitator, but the community is actually the expert”.

Health and mental health professionals discussed the importance of honoring choice and autonomy of the parents and families served. They discussed choice and autonomy in terms of supporting a person’s “ability to make choices, to see their family and feel respect, self-respect” and actually asking parents what they need: “the biggest thing we’re doing right now to understand what each household needs is just literally just asking them, what do you want, what do you need”. Honoring a family’s autonomy to know what they need was also linked to promoting empowerment:

*Empowering people to change their situation, and not even just offering resources and referrals but actually like giving people the autonomy and finding their own strength in their own drive and motivation to change it change their situations, I think that’s a really interesting approach and something I’m really excited about doing.*

Organizational-Level Themes. Organizational subthemes included the balance between work, compensation, and protected time, the availability of resources and opportunities available for organizations and health and mental health professionals, system fragmentation, and system and provider support.

Workforce Support. Many participants discussed feeling overwhelmed and burned out, which contributed to high staff turnover rates. In turn, high turnover rates impaired rapport and trust building with families. Major contributors to lack of balance included high caseloads, long hours, low pay, and rigid or unpredictable schedules that negatively impact the quality-of-service delivery and prevent health and mental health professionals from taking time off and caring for personal health and mental health needs. One provider described their experience as needing:

*…a lot of heart and soul, you can’t just do this job for the paycheck. And that’s where a lot of the burnout comes out too, because we want to do the best for our families. You can’t when you are giving me so many cases.*

Health and mental health professionals described the system as “overwhelmed” and not “created for the volume [of clients] that we deal with each and every day”. This included competing high-priority tasks and managing high-pressure, major stressful life events with limited time and supports. These expectations were labeled as overwhelming and unreasonable. In addition, several participants described the impact of vicarious trauma, or the secondary stress resulting from the traumatic experiences of others, and how it affects health and mental health professionals’ wellbeing, and consequently, efficacy or quality of work with clients.

Health and mental health professionals recognized the importance of a positive work environment to support both health and mental health professionals and families, suggesting that “If you’re positive in your environment then the families you help will receive that positivity too”. Engaging in self-care, stress-relief, and coping strategies for staff in high stress positions was recommended for supporting the workforce and reducing the secondary stress that results from client primary stress. Such events were described as ways to connect with peers and feel appreciated. Health and mental health professionals also indicated a need for protected time for professional development that allowed them to step away from daily responsibilities rather than requiring them to juggle both at the same time. A supportive environment was often linked to support among peers, such as those that encourage taking care of self and family or connecting with co-workers in non-work-related social settings.

Strategies to support health and mental health professionals included a need for additional professional training opportunities in management and leadership and skills for direct practice. Management and leadership training areas discussed focused on supervision of and support for employees, promotion of self-care, communication, and recognizing and addressing secondary stress. Direct practice training and professional development needs included areas such as active listening, motivational interviewing, cultural competence, and addressing complex trauma or co-occurring traumatic events.

*As I know for myself, we’re not trained to be caring, supportive managers. It’s not my background, but it’s not most people’s backgrounds. Social work gives you a background that would be supportive and understanding and put systems in place. Most of us, we train people in management, and what they say is we need more training in management because it’s not enough. And its huge hiring people, training, supporting, and maintaining that support and self-care.*

Resources and Opportunities. The resources and opportunities subtheme refers to financial, logistical, or infrastructure factors impacting organizational functioning or service provision. Health and mental health professionals talked about not having enough money to support important child and family programs, including school programs, social programs, programs for upkeep of community parks and recreational areas, or to pay a fair wage for attracting, hiring, and retaining staff with relevant qualifications. Health and mental health professionals felt ill-equipped in terms of financial and practical resources to meet the level of client need in their agencies and communities. Resource limitations were a salient challenge for smaller and grassroots organizations due to the costs of maintaining adequate physical space. For example, a provider stated: “*So that has been a huge stressor and we’re trying to combat that by hiring more people, but I think it just comes down to the income thing too*”.

Coordinated Care. Several health and mental health professionals described the need for a coordinated system of social care and one that helps families access the important social care services they need rather than relying on a fragmented web of services designed to support the same network of children and families.

*The social service systems really aren’t about helping the family, they’re about identifying what the problems the family has are and then referring to some other system to solve the problem. …. And we wonder why kids keep coming back into the system, or why their poverty doesn’t get addressed, or why the mental health issues of the parents are not addressed, or even of the children. And that’s because there is no integration between these departments to take a holistic approach to the family and a holistic response to their needs, we don’t do that. At least in my experience in the health and human service system.*

Several health and mental health professionals shared the strategies they use to respond to the fragmented systems that create major barriers for families needing or receiving services. One provider described a program developed to provide families with unified support, separate from any existing major institutions: “*and you can build services in…but if they’re all in the same building and they’re all getting their referrals from a team of social workers who are not dedicated to just one system, you can- that works*”. Other health and mental health professionals described strategies used by multiple programs to organize and enhance collaboration and service delivery. These approaches include adjusting the timing of programs to better meet the needs and schedules of families and children, breaking problems into manageable parts, and building on smaller partnerships within the community, recognizing that service goals were interrelated with other issues:

*Food security just doesn’t exist in a vacuum and people who are food insecure have other challenges in their life and you’re never going to get someone sustainable access to food, without addressing some of their other challenges as well.*

#### 3.2.3. Family Level

Subthemes under the family level included supporting parents and the family system and attending to complex needs or supports for co-occurring unmet basic needs.

Support Parents. Health and mental health professionals talked about support that could help families navigate large and complex community institutions, services, and programs, including increased awareness of the resources that are available and how to navigate them. Empowerment of children and families was recognized as an essential element for success.

*And so navigating services I think is very challenging for our families, trying to access support. I think we throw around the word support and we say, “oh here goes a resource”. But how do we teach parents to get to that resource? And so- and even children, we can throw [sic] things at them all day, but actually sitting down to teach them how to access it and how it can be beneficial to them, I think we do a horrible job. Which then creates pressure and folks are just not having the knowledge to empower them to make informed decisions.*

Health and mental health professionals also discussed factors and strategies that support access to services. This included outreach efforts to help families apply for government support programs, consistent and convenient availability of health and mental health professionals, and providing long-term, rather than short-term, services to support the “long-haul work” required in some cases. Expertise of health and mental health professionals and legal advocates was also noted as an important element to navigating bureaucratic systems such as courts and public assistance and especially important in promoting housing stability by preventing evictions.

Several health and mental health professionals discussed the need for programs that are inclusive of or cater to modern family structures, specifically fathers, while still recognizing the struggle for mothers who frequently bore sole or majority responsibility of children.

*So, it’d be nice to see a program where a father could have more concrete supports, if he could take the kids into a drug treatment center and have father-focused programs too. There’s so much weight [sic] on the mother, we need to have some more supports for dad.*

Several health and mental health professionals discussed the importance of having reliable, convenient transportation as well as reliable, trustworthy childcare for facilitating access to needed resources, services, and employment. Although public transportation was discussed as an option, it was widely recognized as problematic for many families, particularly with children in tow.

*Also, just in terms of transportation like it’s hard to get on the bus to go to the supermarket with five kids and one of them has autism like it’s a nightmare. On top of not having the money and like being limited by choice and being limited by like what you get.*

Support Mental Health. Health and mental health professionals discussed parent and child mental health as an important support factor. There was a clear need to expand and deepen patient–provider trust and re-assess and re-establish the client’s confidentiality. Culturally appropriate, high-quality services that accept Medicaid insurance coverage and do not have a long waiting list were consistent issues. Intergenerational trauma, mental health stigma, and intersections of mental health and child welfare system involvement were also consistent issues raised. Participants believed that if more children and families were able to access culturally appropriate services, including trauma-informed care and alternatives to traditional talk therapy, without the stigmas associated with mental illness and substance use, opportunities for reducing disparities would be significant.

*Like, I would love if there were more health mental health resources available for families for specialty therapies like play therapy, music therapy, art therapy. Especially children would benefit from it so much. …. But therapy- consistent therapy would help them understand a better opportunity, a better way, I believe.*

Economic Stability. Unmet basic human needs were discussed primarily in reference to food and shelter in the context of poverty. Housing was identified as a major challenge. This included discussion of affordable living conditions devoid of toxins like lead and mold. Health and mental health professionals discussed the importance of housing in healthy growth and development of children as well as quality of life for caregivers. One service provider commented that “the toughest challenge right now is housing, that’s where we find the most difficulty because we have a difficult time finding assistance. And that you know, stunts their growth”. Another provider commented:

*And the challenges that I think most often affects our families, currently- and we have a hard time looking for this kind of support is- housing is, because of inflation, it’s caused such a hardship on families as far as housing, I mean … rent has gone up exponentially.*

Poverty is cited as “the common denominator” that can give rise to other challenges for many families. For example, families living in poverty “don’t earn enough money to care for the household and the children…and they don’t have that ability to take off [to receive access to services], so they’re losing money”. Further, living in a community with high concentrations of poverty increased chances that the family’s social network also experiences high levels of financial strain, limiting the availability of informal supports.

Health and mental health professionals identified major sources of family stress. This included lack of access to quality education, living wage employment, employment with a stable or reliable schedule, decent housing, and adequate and affordable sources of nutrition for children and families. Other commonly needed day-to-day items included formula, feminine hygiene products, and small amounts of cash for emergencies.

*We really have to get serious about family stability through like economic support. Like I just think about like we invest so much in surveilling controlling and separating poor families, what if we spent that money on actually providing economic stability.*

Intersecting Support Needs. Many health and mental health professionals discussed the need for interventions that address the intersecting priorities of families. For example, health and mental health professionals discussed the high probability of multiple, intersecting traumas experienced by a single family within a short time frame, such as witnessing or experiencing gun violence or interpersonal violence (e.g., domestic violence, child abuse or neglect), while managing major financial stressors, acute or chronic mental or physical health conditions, and system-level threats that prevent access to needed services in a non-stigmatizing and non-oppressive manner. This was particularly challenging when families were in crisis and survival mode. As stated by one service provider:

*So, it’s a lot of different things I think that can take a toll on them and it’s just too much for them to handle I think. And I think that just mentally just triggers them to either give up or just, you know, either be overwhelmed or, you know, depressed.*

## 4. Discussion

Examination of in-depth, semi-structured interviews with administrators and health and mental health professionals from child and family-serving CBOs, guided by an ecocultural conceptualization, revealed a full range of long-standing factors that could facilitate improvements to child and family health/mental health in under-resourced communities in a large metro area. For example, our results suggest child and family functioning are influenced not only by individual psychological, physiological, and cognitive development processes of the clients but also by various interconnected environments, such as family, peers, childcare resources, communities, and community organizations. Within an ecological systems framework, our results represent the multidetermined processes associated with client access and engagement in social services programs and the influence of these factors on program effectiveness and health promotion outcomes. While many of our data support existing evidence of persistent challenges, novel takeaways from the ecological assessment approach highlight key factors/determinants of health outcomes in low-income communities across four broad ecological levels–evidence that could guide multilevel strategies to improve community capacity, clients’ uptake and engagement in social services programs, and program effectiveness on early childhood health outcomes.

Novel study findings were derived from participant perceptions emphasizing the primacy of addressing common underlying determinants of poverty and oppression while reinvesting in communal cohesion and trust and building organizational capacity to enhance system-wide coordination and accessibility. Other participant perceptions of barriers to health promotion across ecological themes were consistent with existing evidence. For example, subthemes that included racial and ethnic disparities in food and housing security, living in poverty, and lacking appropriate mental healthcare parallel evidence of these barriers in the existing literature [31,32,33,34,35]. Likewise, concerns over community safety, neighborhood cohesion, and upkeep of the physical infrastructure continue to have associations with poorer health outcomes [36,37,38,39,40,41]; and organizational factors, such as quality and frequency of supervision, caseload size, and staff compensation, play an important role in supporting the workforce and preventing work-related stress [42,43,44,45,46]. In addition, family-level barriers related to childcare, transportation, housing, mental health, and time constraints undermine access to and engagement in supportive community programs and resources [47,48].

In the current study, health and mental health professionals frequently described a disconnect between client efforts to change behaviors and promote health and their access to resources necessary to realize these changes available within their communities. Unhealthy behaviors can often be mechanisms for coping with overall stress and poverty-related stressors, and individual-focused interventions in community settings often overlook broader determinants [49,50]. For example, an intervention to increase access to diabetes education demonstrated improvement in nutrition knowledge and cooking skills, but participants were limited in their ability to eat healthier due to affordability of food and supplies in their community [50,51], suggesting that a key underlying problem may be less related to clients’ health-related knowledge and motivation to change than to systemic issues that undermine access to or engagement in healthier behavior.

Health and mental health professionals in the current study suggest that policy efforts focused on systemic change addressing poverty at the macro level are lacking, specifically affordable housing, food insecurity, child welfare, and mental health, services that could benefit from more coordinated models of care. The participants further described a lack of policies and structures that address these underlying issues of poverty, consequently undermining their ability to help families in crisis. Findings support the assertion that advocacy should focus on improving policy related to social services and anti-poverty programs, with a particular focus on including low-income, marginalized populations in the decision-making process [52]. Overall, existing evidence demonstrates little focus on upstream policies related to common underlying determinants of health such as poverty and housing [53] that could potentially be better addressed by coordinated system interventions. However, the feasibility of such systems is complex and challenging to implement due to the extensive cross-sectoral collaboration, leadership, and resources required.

Many of the macro-level factors discussed by participants have a strong association with racism and oppression. Consistent with the Centers for Disease Control and Prevention (CDC), people of color are more likely to perceive their neighborhoods as unsafe, as well as feel disconnected from efforts to improve health in their communities [54]. Families of color are also more likely to experience housing and food insecurity [31,32], live in poverty [33], and lack access to mental healthcare [34]. Additionally, consistent with concerns raised by participants in this study, research has routinely demonstrated disparate outcomes across all levels of the child welfare system for families of color [35]. In considering how public health and social service efforts can build better health and mental health equity across communities and organizations, study findings suggest it is important to understand the barriers faced by marginalized communities and center the voices of communities experiencing racism and poverty.

Consistent with prior research, participants pointed to the physical infrastructure of a neighborhood and opportunities to build social networks as important community-level themes. Community risk factors such as concerns over physical safety, low neighborhood cohesion, and other detracting neighborhood elements (e.g., litter, dilapidated housing, vandalism) have been consistently linked to poorer health and wellbeing [36,37,38]. Limited access to recreational facilities limits outdoor play and negatively impacts physical health [39,40,41]. When families have access to recreational centers and safe, clean, green spaces, they have more autonomy and control over recreational activities and are more likely to engage in them [55]. In addition to physical infrastructure, cohesive, safe, and trustworthy social networks can be an effective buffer between stressful conditions and physical and mental wellbeing by shaping perceptions of community safety and reducing fears of safety risks [38,56].

A common organizational-level theme discussed in the current study was the need for supports that address secondary stress and burnout within community health agencies which, in turn, can affect program implementation and effectiveness. Organizational factors such as quality and frequency of supervision, caseload size, and staff compensation play an important role in service provider stress prevention [42], as do organizational supports for employees [43]. In addition to work–life balance, health and mental health professionals in the current study discussed the need for additional professional development for employees. Professional development is an opportunity to not only improve the services provided to clients but to also improve positive coping mechanisms among employees frequently exposed to client trauma, particularly self-care, which can reduce burnout [44,45]. Professional development of health and mental health professionals should also focus on community engagement and empowerment to facilitate service uptake [57]. Further, lack of knowledge about trauma and available trauma treatment can undermine African American families’ access to mental healthcare, as can fears of being reported to child welfare and legal systems. Because mental healthcare was cited as a significant unmet need, consistent with the prior literature, refs. [58,59], workforce training on trauma-informed approaches to working with high-risk populations maybe important [5].

Importantly, participants in the current study noted the negative impact of fragmented services, including a disconnect between organizations and families, on the quality and effectiveness of program implementation and effectiveness. Carey [60] noted that fragmentation of social services negatively impacts clients, as well as health and mental health professionals, and interferes with relationship development in social services. Coordinated care efforts show promise in both health and mental healthcare [61,62]; however, there is a dearth of coordination in existing social care networks. Future efforts should focus on promoting and evaluating coordination of social services to promote health equity among children and families in low-income communities.

Health and mental health professionals in the current study were working with families with complex needs across multiple, often co-occurring areas: basic needs (food and shelter), resources (income, transportation, and childcare), and health (mental health and trauma), commonly known challenges that intensified during and after the COVID-19 pandemic. Families frequently struggled with having adequate income and financial resources to meet their health and wellbeing needs. Access to gainful employment that pays a living wage is hard to access for low-income families, especially those with young children. Health and mental health professionals noted poverty as a driving force for why families needed CBO services. Services were often difficult for families to access due to an inability to take time off work and the inconvenient timing and location of services. Insufficient transportation and childcare resources were other common and longstanding barriers to accessing needed services. Evidence consistently indicates that both childcare and transportation play a pivotal role in accessing needed health services [47,48]. Additional research on transportation and childcare interventions as well as system coordination to increase convenience of service location to reduce service access barriers for families and children is needed. Another area of unmet need discussed by interview respondents was around mental healthcare, especially trauma-specific care, which they noted was often minimized or ignored in discussions with families. Consistent with prior research, discussion highlighted clients’ cultural preferences, favoring faith and religious community support over mental healthcare as a source for healing during traumatic experiences among African American families [63,64].

The current study contributes to our understanding of frontline health and mental health professionals’ perspectives of barriers to and promoters of health equity among urban service users. A key strength of this study is that it includes the perspectives of those working in complex and often fragmented social service systems in overlapping metropolitan communities affected by a complex, multidetermined ecology. Thus, perspectives from these health and mental health professionals offer insight into areas of potential improvement. This study is rooted in the ecological systems perspective as a guiding framework throughout. Within the ecological systems perspective, there is the important understanding that health and mental health professionals, policy makers, and researchers cannot expect change from our clients without working with them to make sure they have access to the services needed to support those changes, such as cohesive and collaborative services that incorporate the strengths of families while recognizing their unique needs. This type of ecological assessment approach can serve as a model for similar types of underserved communities in which multiple, fragmented social services and health promotion agencies could coordinate efforts to (a) assess varying perspectives across a range of community-, organizational-, and family-level determinants and (b) use such formative analysis to guide community-specific coordinated programs and services that better address the interacting factors across levels of influence that perpetuate access and quality of care deficits in their individual programming and implementation efforts.

There are also limitations that must be noted. As the cross-sectional study is qualitative in nature, it cannot be generalized beyond the sample population or over time: health and mental health professionals in one CBO system serving a large, persistently impoverished urban area. The possibility of selection bias is also a potential limitation, as participants were primarily female and more heavily represented certain service areas, such as child welfare and food assistance, while fewer participants were recruited from mental and behavioral health services. Although we employed purposive and snowball sampling strategies to capture diverse perspectives, a broader, longitudinal representation of health professionals across various service sectors and over time may be useful for enriching findings of future studies in this area. Although attempts were made to monitor for and address researchers’ bias, as is recommended for qualitative research, it is important to note that the present study and the interpretation of the data collected, are presented through the lens of the researchers, and this is subject to their biases and experiences. Additional research on this topic is necessary.

## 5. Conclusions

### 5.1. Theoretical Implications

Novel study findings grounded in ecological framing recognize the primacy of addressing common underlying determinants of poverty and oppression while reinvesting in communal cohesion and trust and building organizational capacity to enhance system-wide coordination and accessibility. These findings point to the utility of a thorough ecological assessment of community health system providers and staff to better understand complex determinants of program effectiveness in persistently impoverished communities to reduce health inequities.

### 5.2. Practical Implications

Ecological assessments could guide agency coalition building and development of multilevel approaches that de-emphasize individual-focused behavior change interventions delivered within siloed settings in favor of social services and behavioral interventions that account for the interactions between individual behavior change and health outcome determinants across the broad ecology in which they are implemented. Better attention to the family-, community-, and policy-level factors that either constrain or facilitate agencies’ efforts to address child and family health inequality would ultimately guide multilevel intervention efforts that include changing the structure of setting themselves to improve program implementation and effectiveness. Major themes in this study point to consistent and novel multilevel implications and the understanding that approaches to prevention and intervention can and should address the interaction of complex needs and support factors across levels of the family’s ecology. In addressing the unique and elevated challenges to health behavior promotion in racially/ethnically diverse and economically disadvantaged populations, multilevel ecological approaches can overcome the limitations of standard treatments [65]. For example, a novel multilevel intervention for low-income maternal smokers initiated in urban clinics delivering the Special Supplemental Nutrition Program for Women, Infants and Children (WIC) was designed to improve cessation treatment access, engagement, and effectiveness by combining a brief tobacco screening and advice and referral intervention delivered by WIC nutrition professionals with a 12-week, multimodal telehealth-based intervention modeled after Quitline infrastructure and designed to address individual- and home-level determinants of smoking [66]. This multilevel intervention was superior to standard care in promoting long-term bioverified tobacco abstinence [67] and provides a translatable multilevel approach to addressing complex health behaviors in low-income communities by combining a trusted low-income community clinic-based, systems-level intervention with a wider reaching and more comprehensive telehealth intervention.

### 5.3. Policy Implications

In addition, improving community capacity to inform pro-health equity legislation, regulations, and policies could strengthen the broader context within which community-based interventions in persistently impoverished areas are implemented. However, we also recognize the complexity of implementing coordinated policy systems that cohesively address systemic issues such as poverty, housing instability, and food insecurity. Such changes would involve navigating existing policy constraints, funding limitations, and inter-agency collaboration barriers. Future research should explore the feasibility of successfully integrated service models and identify specific policy mechanisms that could facilitate large-scale implementation. At the community level, workforce training on cross-sector collaboration for systems of organizations in the child and families sector could improve community- and organizational-level relationships that better address underlying social determinants of health and ways in which trauma and poverty interact through community-level factors to influence patterns of healthy child development [5].

## Figures and Tables

**Table 1:** Ecocultural family interview global topics and sample probing items.

Topic Areas	Sample Items
Home Environment	What are the barriers or stressors affecting parent and child health and mental health? Why? Which are most pressing? What about accessing the needed health or mental care they need? How do you think major barriers or stressors affect parent and child health behaviors like eating “healthy” and food purchasing behaviors and foods available at home?
2. Family Health	In what ways do families protect their health? What steps have you seen families take? In what ways do you think you in your role help facilitate or contribute to the health and wellness for families, parents, or kids? Others in your role, your organization, other organizations/agencies like yours? What resources or services would you recommend to a parent to help them cope with stress? What about resources or services for their kids?
3. Organizational-Level Challenges and Facilitators	From your perspective, what do you think are the top challenges currently facing- you in your role, others in your role, your organization and leaders in your organization, other organizations like yours, parents, caregivers, and children? What major challenges or stressors do you think have impacted staff and client relationships? How do you think these major challenges or barriers affect the health and wellbeing of children and families?
4. Community Supports and Services	What kind of support do you and people in your role have for dealing with challenges or barriers? From the organization? Their community? What do you think is needed to strengthen programs to better serve families and communities? What resources or services would you recommend to a parent to help improve their physical health? Their mental or behavioral health?

**Table 2:** Sample demographics.

Sample Characteristics	*n*	%	*M*	*SD*
Gender—Female	17	81		
Service sector				
Child Welfare	8	38		
Child and Family Programs ^a^	6	29		
Food Assistance	5	24		
Mental/Behavioral Health and Substance Use	2	10		
Role				
Leadership/management	9	43		
Direct practice	12	57		
Educational Background				
Social Work	4	20		
Education	4	20		
Human Service/Social Science	7	33		
Public Health	1	5		
Other	5	24		
Race/ethnicity				
White	6	38		
African American	7	44		
Black	1	6		
Hispanic	1	6		
Asian/White (Mixed)	1	6		
Age				
31–40	11	59		
41–50	4	21		
51+	4	21		
Years in the Field (1–44)	19		13.47	12.33
Years in Current Position (1–29)	20		6.4	7.067

^a^ Child and family programs capture and an array of non-system-related resources and programming for children and families: parent support programs, social and emotional development programs for youth and families, child and family comprehensive care services, legal advocacy, community–family engagement specialists. Note: *n* represents the number of respondents indicated for each item; some items (years in field/current position) are <21 due to missing data.

**Table 3:** Primary themes, subthemes, and definitions.

Theme Title	Definition
1. Macro Level	Things that focus on how broader social, cultural, and political factors affect child and family health and wellbeing.
Rights-based policy/programming	Government- and organizational-level policies and programs related to basic human rights (e.g., food and shelter) that support child or family health and wellbeing.
Racism/oppression	Policies, programs, or practices that alienate, stigmatize, or oppress racial and ethnic minority populations.
2. Community Level	Things that involve the formal and informal social structures directly impact the child-/family-serving organizations that indirectly operate to impact families.
Physical environment	Physical structures, features, and facilities that make up the environments in which people live, work, or interact; the ways in which communal spaces are structured and used.
Building community	Factors that support health or facilitate access to health-promoting resources related to community engagement practices, partnerships, wrap-around supports.
3. Organizational Level	The system of interlinked organization level systems that interacts with the family level to impact child or family health/wellbeing.
Work–life balance	Lack of balance between work, personal life, and pay.
Resources and opportunities	Financial, logistical, or infrastructure factors impacting organization-level functioning or service provision.
Coordinated care	System fragmentation or degree of coordination among organizations serving the same network of children and families.
4. Family Level	Things that have direct contact with the child and their immediate, proximal environments.
Support parents	Factors that identify areas of support for child and family health, including financial stability, access to facilitators like transportation and childcare, and promoting parent/child choice and autonomy.
Support mental health	Factors that identify areas of support for child and family mental health, access to quality and affordable mental health services, and increasing the cultural relevance of mental health services provided.
Economic stability	Factors related to unmet basic needs and access to resources such as a stable income, gainful employment, food, and housing.
Intersecting support needs	Addressing intersecting support needs such as mental health and economic stability.

## Data Availability

Data are unavailable due to privacy/ethical restrictions as per the IRB protocol due to the risks associated with breach of confidentiality in relation to the nature of qualitative research with small sample sizes.

## Abbreviations

The following abbreviations are used in this manuscript:

CBO	Community-Based Organization
EFI	Ecocultural Family Interview
IRB	Institutional Review Board
MSW	Master of Social Work
